# Multi-chamber Intracardiac Extension and Embolic Stroke: A Rare Complication of Mediastinal Non-seminomatous Germ Cell Tumor

**DOI:** 10.7759/cureus.105542

**Published:** 2026-03-20

**Authors:** Waleed Dawelbeit, Marwa Makhloof, Asma Atfeesh, Yasir Almalki, Ahmed Basuoni

**Affiliations:** 1 Cardiology, Cardio-Oncology Unit, Medical Specialties Department, Sultan Qaboos Comprehensive Cancer Care and Research Center, Muscat, OMN; 2 Hematology, Sultan Qaboos Comprehensive Cancer Care and Research Center, Muscat, OMN; 3 Clinical physiology, Clinical Physiology Unit, Medical Specialties Department, Sultan Qaboos Comprehensive Cancer Care and Research Center, Muscat, OMN

**Keywords:** embolic stroke, germ cell tumor (gct), intracardiac tumor extension, malignant pericardial tamponade, medical debulking, superior vena cava obstruction (svco), transesophageal echocardiography (tee), vip chemotherapy protocol

## Abstract

Primary mediastinal non-seminomatous germ cell tumors (PMNSGCT) are aggressive malignancies. A rare and high-risk complication is the extension of the tumor into the cardiac chambers or the formation of tumor-associated thrombi, which carry a profound risk of systemic or pulmonary embolization.

We present a complex case of a 21-year-old man with superior vena cava obstruction (SVCO) and massive, multi-chamber intracardiac tumor extension discovered incidentally following a presentation of chest pain and ST-segment elevation. He was found to have a pericardial effusion that later progressed to impending tamponade. Transesophageal echocardiography (TEE) revealed highly mobile masses within the right atrium (RA), right ventricle (RV), and left atrium (LA), the latter originating from the pulmonary veins.

The clinical course was complicated by embolic cerebral infarcts. Despite the high risk of further embolization, the patient was managed with emergent pericardiocentesis, therapeutic anticoagulation, and VIP (Etoposide, Ifosfamide, Cisplatin) chemotherapy protocol. The patient achieved complete resolution of the intracardiac masses, demonstrating the efficacy of the “chemotherapy-first” medical debulking approach in managing high-risk inoperable intracardiac tumor burden.

This case highlights the important role of echocardiography in intracardiac tumor diagnosis and the clinical challenge of distinguishing tumor thrombus from direct extension. It demonstrates the efficacy of a chemotherapy-first approach in managing high-risk or inoperable intracardiac tumor burden.

## Introduction

Primary extragonadal malignant germ cell tumors (GCTs) are rare, accounting for only 2%-5% of the malignant germ cell tumors [1]. Intracardiac extension of extragonadal GCTs represents an extreme oncological emergency [2]. Although a rare phenomenon, these tumors typically infiltrate the heart via two distinct pathways: direct transmural invasion or endovascular growth through the vena cava or pulmonary veins. Patients are at risk of developing severe, life-threatening complications related to intracardiac invasion, occurring either in isolation or in combination, including systemic or pulmonary embolization [3-5], pericardial effusions with tamponade [6], or mechanical obstruction of cardiac valves resulting in haemodynamic collapse. Managing such cases is particularly challenging when the tumor burden is deemed surgically inoperable due to the extent of the cardiovascular involvement. This case describes our multidisciplinary approach to a complex GCT with multi-chamber intracardiac masses, further complicated by local and distant embolic phenomena.

## Case presentation

A 21-year-old man with no significant medical history presented in April 2022 with progressive dyspnea, orthopnea, and clinical signs of superior vena cava obstruction (SVCO). Imaging revealed a large infiltrative hypodense solid mass lesion in the anterior mediastinum measuring approximately 13×10 cm. The lesion was inseparable from the aortic arch and its major branches in the superior mediastinum. A subsequent biopsy confirmed a primary mediastinal non-seminomatous germ cell tumor (PMNSGCT) with yolk sac and embryonal carcinoma elements.

**Figure 1:**
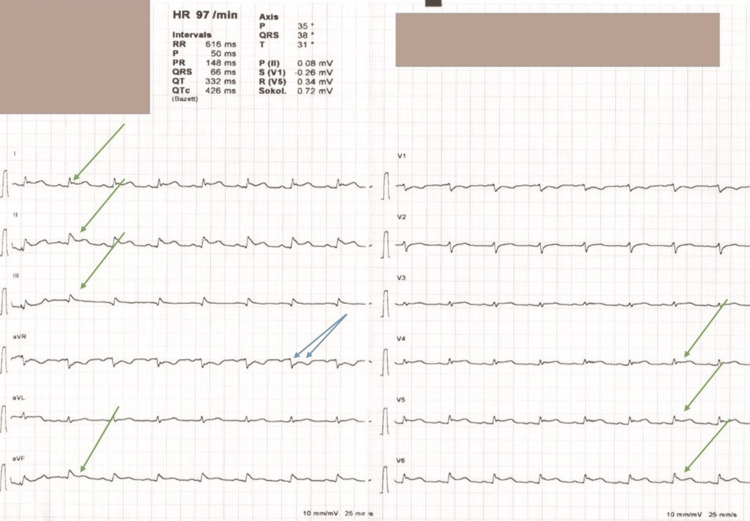
Twelve-lead electrocardiogram (ECG) showing diffuse ST-segment elevation The 12-lead ECG demonstrates widespread ST-segment elevation across the precordial (V4-V6) and limb leads (I, II, III, and aVF). Reciprocal ST-segment depression in lead aVR. These findings are consistent with acute pericarditis. Single green arrows highlight the ST-segment elevation, while double blue arrows denote the reciprocal changes in lead aVR.

During admission, the patient developed severe chest pain. An initial electrocardiogram (ECG) showed ST-segment elevation in inferior leads and V4-6, raising suspicion of an embolic myocardial infarction (Figure 1).

Bedside echocardiography revealed a pericardial effusion and highly mobile masses within the right and left atria and attached to the tricuspid valve (TV), raising a differential diagnosis of vegetations, thrombi, or direct tumor extension. However, the absence of regional wall motion abnormalities (RWMA) on echocardiography and the high-risk nature of the cardiac masses shifted the diagnostic focus toward severe pericarditis and the embolic risk.

**Figure 2:**
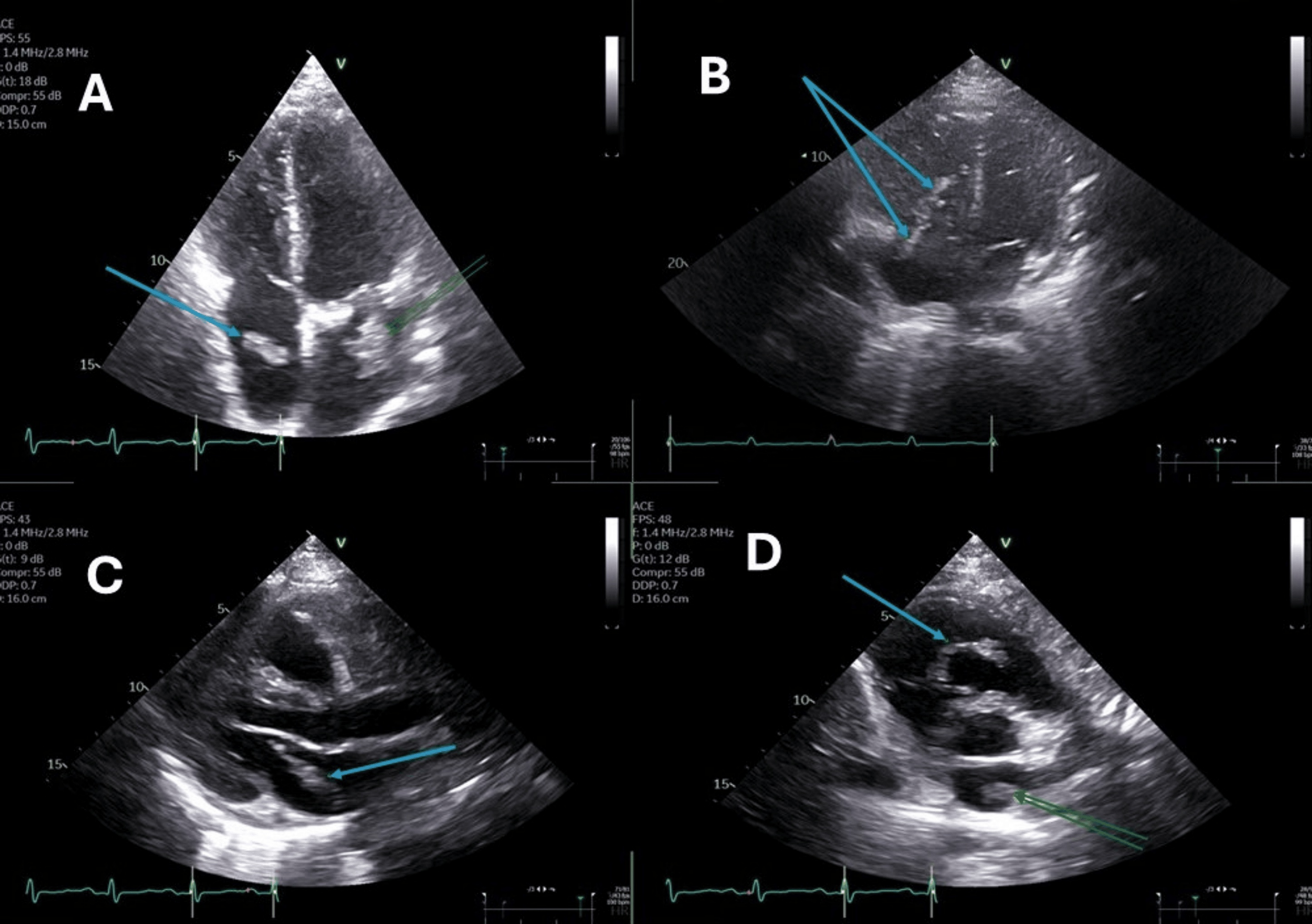
Transthoracic echocardiography (TTE) of bilateral cardiac masses A: Apical four-chamber view (end-systole). Single blue arrow indicates the right atrial mass; double green arrows indicate the left atrial mass. B: Apical four-chamber view (diastole). Double blue arrows highlight the right atrial mass prolapsing from the right atrium into the right ventricle across the tricuspid valve. C: Parasternal long-axis view (diastole). Single blue arrow identifies the left atrial mass. D: Parasternal short-axis view (end-diastole). Single blue arrow shows the right atrial mass in the right ventricular outflow tract; double green arrows indicate the left atrial mass.

Two days later, the effusion increased, leading to clinical features of impending tamponade. Repeat echocardiography demonstrated right atrial (RA) and right ventricular (RV) diastolic collapse, mitral valve inflow respiratory variation, and a plethoric inferior vena cava (IVC) (Figure 2, Video 1).

**Video 1:** Transthoracic apical four-chamber view showing bilateral masses A looped transthoracic echocardiogram (TTE) in the apical four-chamber view demonstrates the dynamic mobility of the bilateral cardiac masses. The single green arrow indicates the right atrial mass, while the double green arrows highlight the left atrial mass.

**Figure 3:**
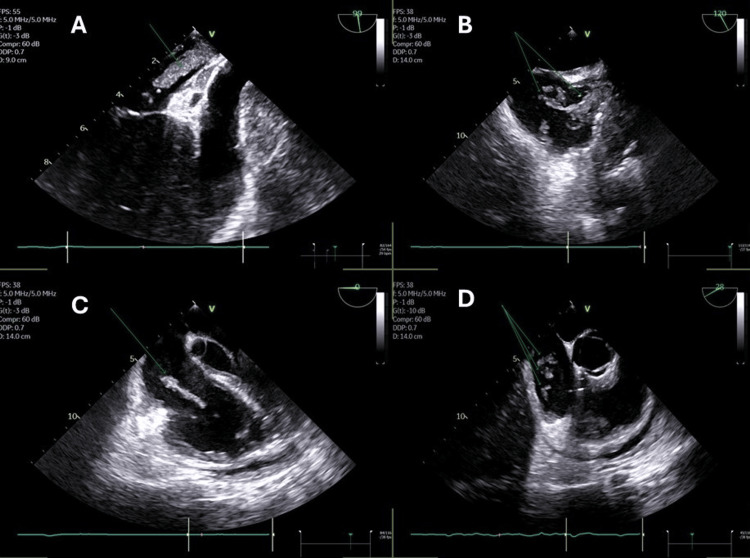
Transesophageal echocardiography (TEE) showing mass origins and mobility A: Mid-esophageal 90-degree view (pulmonary vein adjusted). Single green arrow identifies the left atrial mass protruding from the left superior pulmonary vein. B: Mid-esophageal bicaval 120-degree view (superior vena cava adjusted). Double green arrows show the right atrial mass emerging from the superior vena cava. C: Mid-esophageal 0-degree view. Single green arrow demonstrates the right atrial mass prolapsing from the right atrium into the right ventricle across the tricuspid valve. D: Mid-esophageal 28-degree view. Triple green arrows indicate the right atrial mass within the right atrial chamber.

Emergent pericardiocentesis drained 750 ml of hemorrhagic fluid (cytology positive for malignant cells). Once stabilized, a transesophageal echocardiogram (TEE) provided detailed visualization of the intracardiac masses. There were two right-sided masses: a serpiginous 32x12 mm mass extending from the superior vena cava (SVC) into the right auricle (RA), protruding into the right ventricle (RV), and highly mobile. Another highly mobile mass was also seen attached to the tricuspid valve. While on the left side, there was a 25x8 mm mass seen in the left atrium (LA), invading through the left upper pulmonary vein, highly mobile, reaching up to the mid-left atrial cavity (Figure 3, Videos 2, 3).

**Video 2:** Transesophageal 99-degree view (pulmonary vein adjusted). A looped transesophageal echocardiogram (TEE) at 99 degrees illustrating the left-sided pathology. The single green arrow identifies the left atrial mass protruding from the left superior pulmonary vein into the left atrial cavity.

**Video 3:** Transesophageal bicaval 120-degree view (SVC adjusted) A looped transesophageal echocardiogram (TEE) focused on the superior vena cava (SVC) inflow. Double green arrows show the right atrial mass emerging from the SVC and prolapsing into the right atrium.

On the fifth day of admission, the patient experienced two episodes of transient loss of consciousness; a brain magnetic resonance imaging (MRI) confirmed subacute embolic infarcts in the right caudate lobe. Given the LA mass background and lack of other risk factors, this was identified as the embolic source. Due to the infiltrative encasement of the great vessels, the patient was deemed surgically inoperable. He was initiated on the VIP protocol (Cisplatin, Etoposide, Ifosfamide) and therapeutic anticoagulation (Apixaban). He showed a dramatic response, with a significant reduction in mass size and resolution of the SVCO. By September 2022, follow-up TEE confirmed complete resolution of all intracardiac masses. In December 2022, he underwent successful surgical resection of the residual mediastinal mass. Long-term follow-up revealed stage IIIa chronic kidney disease, a late sequela of platinum and Ifosfamide-based chemotherapy toxicity [7].

## Discussion

This case highlights the aggressive nature of PMNSGCT. Intracardiac involvement often represents a hybrid of direct tumor extension and secondary thrombus formation. The presence of a mass in the pulmonary vein extending into the left atrium is a direct cause for cerebral infarction, while right-sided heart masses pose a significant threat for pulmonary embolism [4,8].

The rapid resolution of these multiple, large intracardiac masses following chemotherapy suggests they were composed primarily of viable tumour cells rather than traditional blood clots. These masses regressed in tandem with the primary mediastinal lesion; however, the lack of a direct biopsy, due to the prohibitive procedural risk, remains a limitation in the pathological characterization of the masses.

While large, mobile cardiac masses often warrant surgical intervention to prevent further embolic events, the infiltrative "encasement" of the great vessels in this patient made cardiac surgery technically prohibitive; Consequently, he was managed with the VIP chemotherapy protocol and therapeutic anticoagulation. VIP has a favorable outcome and survival in patients with poor-risk germ cell tumours [9,10].

This case highlights the clinical challenge of distinguishing tumor thrombus from direct extension. It underscores the efficacy of a “chemotherapy-first” or “medical debulking” approach in managing high-risk or inoperable intracardiac tumour burden, potentially avoiding the morbidity of open-heart surgery in a hemodynamically fragile patient. It also demonstrates the diagnostic utility of TEE in characterizing intracardiac masses.

## Conclusions

Multi-chamber cardiac invasion by GCTs is a critical oncological emergency requiring a rapid, multidisciplinary approach. A transesophageal echocardiogram is essential for the precise visualization of the superior vena cava and the pulmonary vein involvement, especially when conventional imaging is inconclusive. Systemic embolization is a high risk with left atrial involvement, necessitating early initiation of therapeutic anticoagulation and systemic chemotherapy if the exact nature of the masses is not determined by a successful biopsy. In cases where infiltrative tumour encasement makes surgery technically prohibitive, this case demonstrates that a "chemotherapy-first" and therapeutic anticoagulation strategy can achieve complete resolution of high-risk intracardiac burdens.
